# Structure-Function Analysis of the *Anopheles gambiae* LRIM1/APL1C Complex and its Interaction with Complement C3-Like Protein TEP1

**DOI:** 10.1371/journal.ppat.1002023

**Published:** 2011-04-14

**Authors:** Michael Povelones, Leanna M. Upton, Katarzyna A. Sala, George K. Christophides

**Affiliations:** Division of Cell and Molecular Biology, Department of Life Sciences, Imperial College, London, United Kingdom; Stanford University, United States of America

## Abstract

Malaria threatens half the world's population and exacts a devastating human toll. The principal malaria vector in Africa, the mosquito *Anopheles gambiae*, encodes 24 members of a recently identified family of leucine-rich repeat proteins named LRIMs. Two members of this family, LRIM1 and APL1C, are crucial components of the mosquito complement-like pathway that is important for immune defense against *Plasmodium* parasites. LRIM1 and APL1C circulate in the hemolymph exclusively as a disulfide-bonded complex that specifically interacts with the mature form of the complement C3-like protein, TEP1. We have investigated the specificity of LRIM1/APL1C complex formation and which regions of these proteins are required for interactions with TEP1. To address these questions, we have generated a set of LRIM1 and APL1C alleles altering key conserved structural elements and assayed them in cell culture for complex formation and interaction with TEP1. Our data indicate that heterocomplex formation is an intrinsic ability of LRIM1 and APL1C and identify key homologous cysteine residues forming the intermolecular disulfide bond. We also demonstrate that the coiled-coil domain is the binding site for TEP1 but also contributes to the specificity of LRIM1/APL1C complex formation. In addition, we show that the LRIM1/APL1C complex interacts with the mature forms of three other TEP proteins, one of which, TEP3, we have characterized as a *Plasmodium* antagonist. We conclude that LRIM1 and APL1C contain three distinct modules: a C-terminal coiled-coil domain that can carry different TEP protein cargoes, potentially with distinct functions, a central cysteine-rich region that controls complex formation and an N-terminal leucine-rich repeat with a putative role in pathogen recognition.

## Author Summary

The malaria-transmitting mosquito, *Anopheles gambiae*, uses a complement-like pathway to defend against *Plasmodium* parasites. The complement C3-like protein, TEP1, binds to the surface of invading parasites, triggering their destruction and clearance. LRIM1 and APL1C, two leucine-rich repeat proteins, form a disulfide-bonded complex which stabilizes mature TEP1 and promotes its binding to parasites. Here, we investigate the structural and biochemical features of the LRIM1/APL1C complex and its interaction with TEP1. We identify key amino acid residues responsible for covalently linking LRIM1 and APL1C and the region of the complex where TEP1 binds. Importantly, we demonstrate that the LRIM1/APL1C complex can interact with the mature form of three other TEPs, including TEP3, which we characterize as a novel *Plasmodium* antagonist. Our results suggest that the LRIM1/APL1C complex has a modular architecture in which distinct functions map to different regions. Our study provides important insights into how the *A. gambiae* complement pathway helps mosquitoes fight against the malaria parasite.

## Introduction

The innate immune system is the primary, and in some organisms, such as insects, the sole means of defense against infection. The main mosquito defense against invading *Plasmodium* is orchestrated by a collection of hemolymph proteins that closely resembles the vertebrate complement cascade [1]. The majority of *Plasmodium* ookinetes traversing the mosquito midgut epithelium and coming into contact with the hemolymph are attacked and cleared by lysis or by encasement in a melanin capsule (melanization). Both of these reactions are triggered by binding on the parasite surface of the thioester-containing protein TEP1, a homolog of the complement factor C3 [2]. The few parasites that escape this reaction develop into oocysts and, protected by the oocyst wall, amplify their numbers and differentiate into sporozoites, the vertebrate infective form of *Plasmodium*.

How parasites are recognized by the mosquito immune system and how complement activation is biochemically regulated remain unanswered, but recent work has revealed that these reactions involve complex networks of basally expressed proteins, including LRIM1 and APL1C, two putative pathogen recognition receptors of the leucine-rich repeat (LRR) immune protein (LRIM) family [2], [3], [4], [5], [6], [7], [8], [9],[10]. LRIM1 and APL1C circulate in the mosquito hemolymph as a disulfide-bonded high-molecular weight complex and are major antagonists of mosquito infections with the rodent parasite *P. berghei* [6]. Silencing the genes that encode LRIM1, APL1C and TEP1 transforms a refractory *A. gambiae* strain into a susceptible strain [2], . Importantly, this triumvirate of proteins contribute to resistance against *Plasmodium* of the non-vector mosquito *A. quadriannulatus* A; their silencing renders these mosquitoes permissive vectors [11]. The LRIM1/APL1C complex interacts with proteolytically processed (mature) TEP1 in the mosquito hemolymph [5], [6]. This interaction stabilizes this mature and reactive form of TEP1, promoting its binding to the parasite surface and preventing its reaction with self.

LRIM1 and APL1C share several conserved structural features including a signal peptide, an LRR domain, a pattern of cysteine residues and a C-terminal coiled-coil domain [6], [12]. LRR domains are common in immune receptors and are flexible in their binding properties, e.g. Toll-like receptors [13] and the variable lymphocyte receptors of jawless vertebrates [14], while coiled-coil domains often mediate protein-protein interactions. The three-dimensional structure of the LRIM1/APL1C heterodimer has been recently determined, revealing the presence of a single disulfide bond between the two proteins formed by conserved cysteine residues and providing a structural framework for elucidation of the function of this innate immune complex [15]. We designed a structure-function biochemical study to further our understanding of the interactions between LRIM1 and APL1C, and to investigate the role of their constituent domains in interactions with TEP1 and other immune proteins. Using a panel of engineered *LRIM1* and *APL1C* alleles we reveal that the cysteine-rich region between the LRR and coiled-coil domains is crucial for LRIM1/APL1C complex formation and corroborate the identity of the cysteines involved in the formation of the disulfide bridge that is however not required for the interaction between the LRIM1/APL1C complex and TEP1. We also show that the coiled-coil domain is largely dispensable for complex formation, but is essential for interactions with TEP1 as well as with three other TEP family members, one of which we show to be a potent antagonist of *P. berghei* infection. Our work reveals a modular nature of the LRIM1/APL1C complex, which is common in LRR-containing innate receptors, and demonstrates that by carrying different cargoes this putative receptor may serve distinct immune functions.

## Results

### LRIM1 and APL1C have an intrinsic ability to form homo- and heterodimers

**Figure 1:**
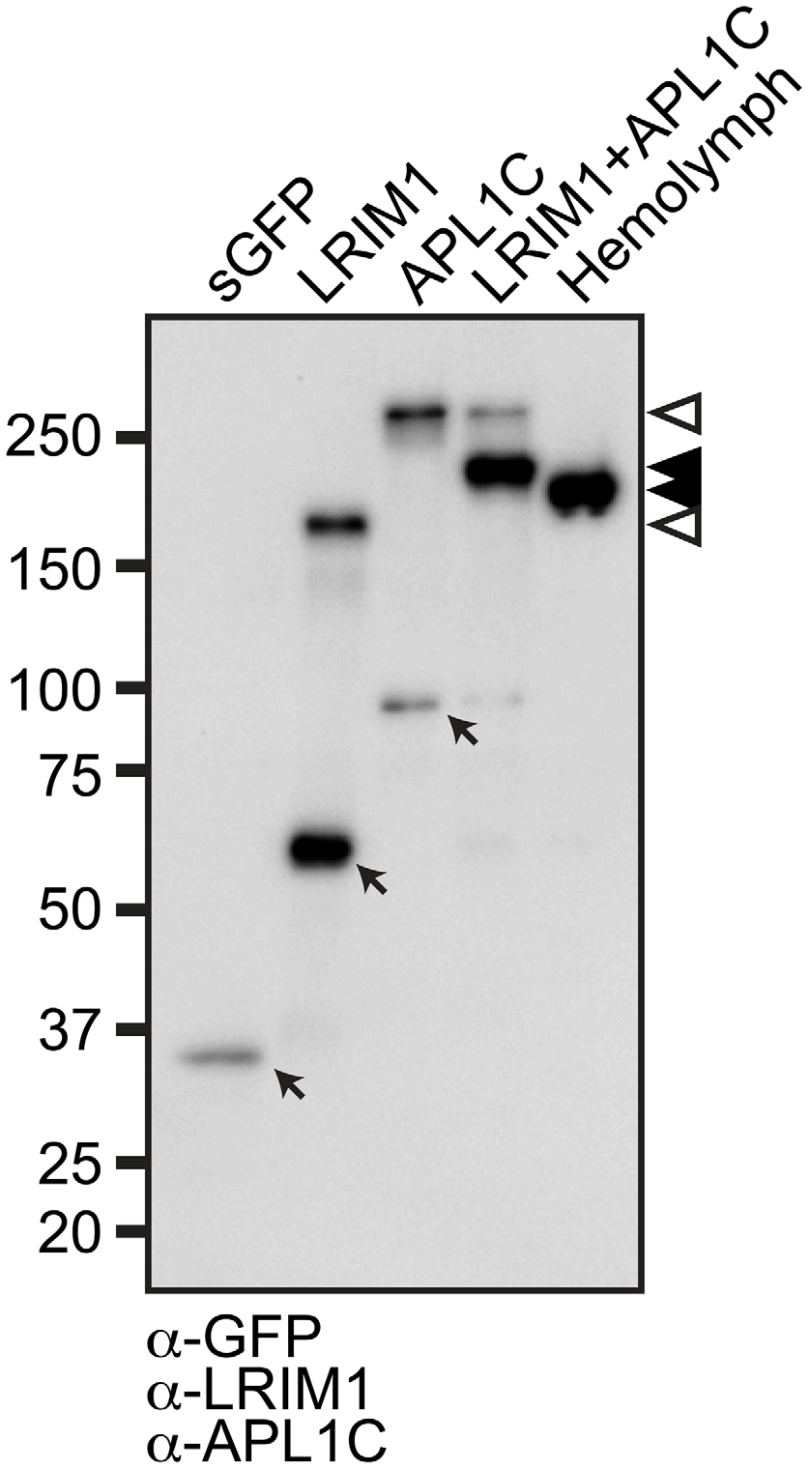
LRIM1 and APL1C form a heteromeric complex. Non-reducing western blot of mosquito hemolymph containing endogenous LRIM1 and APL1C and CM collected from Sf9 cells transfected with tagged LRIM1, APL1C or a secreted GFP (sGFP). The blot was probed simultaneously with antibodies against LRIM1, APL1C and GFP. Indicated are Arrows: GFP, LRIM1 and APL1C monomers. Arrowheads: LRIM1 and APL1C homodimers, white; LRIM1/APL1C heterodimers, black.

The LRIM1 and APL1C proteins circulate in the mosquito hemolymph as a disulfide-linked complex. To determine whether LRIM1 and APL1C have the intrinsic ability to form complexes or if they require a cofactor, we expressed *LRIM1* and *APL1C* alone or together in Sf9 cells, an insect cell line derived from the lepidopteran, *Spodoptera frugiperda*. Given that the LRIM family is mosquito specific, a non-mosquito derived cell line should lack potential interacting partners. For these experiments and those described below we used expression constructs containing *LRIM1* and *APL1C* transgenes that incorporate Strep and His epitope tags on the N- and C-termini, respectively [6]. Following transfection, conditioned medium (CM) was collected from the cells and analyzed by western blot of non-reducing (NR) sodium dodecyl sulfate polyacrylamide gels (SDS-PAGE) using antibodies against LRIM1 and APL1C. CM collected from cells transfected with *LRIM1* or *APL1C* show that each protein individually has the ability to form a high-molecular weight homomeric complex (Figure 1). Co-transfection of *LRIM1* and *APL1C* together preferentially yields a complex intermediate in size compared to their single transfections. As expected due to the presence of the tags, the LRIM1/APL1C heterocomplex is slightly larger than the untagged native complex present in the mosquito hemolymph. Given the observed sizes of the monomeric tagged LRIM1 and APL1C (63 and 97 kDa, respectively) the size of the complexes produced by single (169 and 235 kDa) and double transfections (197 kDa) best fit with homotrimers and heterotrimers. However, the stoichiometry of the LRIM1/APL1C complex was recently determined to be a heterodimer using techniques that are not influenced by protein shape [15]. The same study also showed that both LRIM1 and APL1C could form homodimers. Therefore, the LRIM1/APL1C complex appears to migrate aberrantly slowly on SDS-PAGE gels as is commonly reported for proteins containing coiled-coils [16], [17], [18].

**Figure 2:**
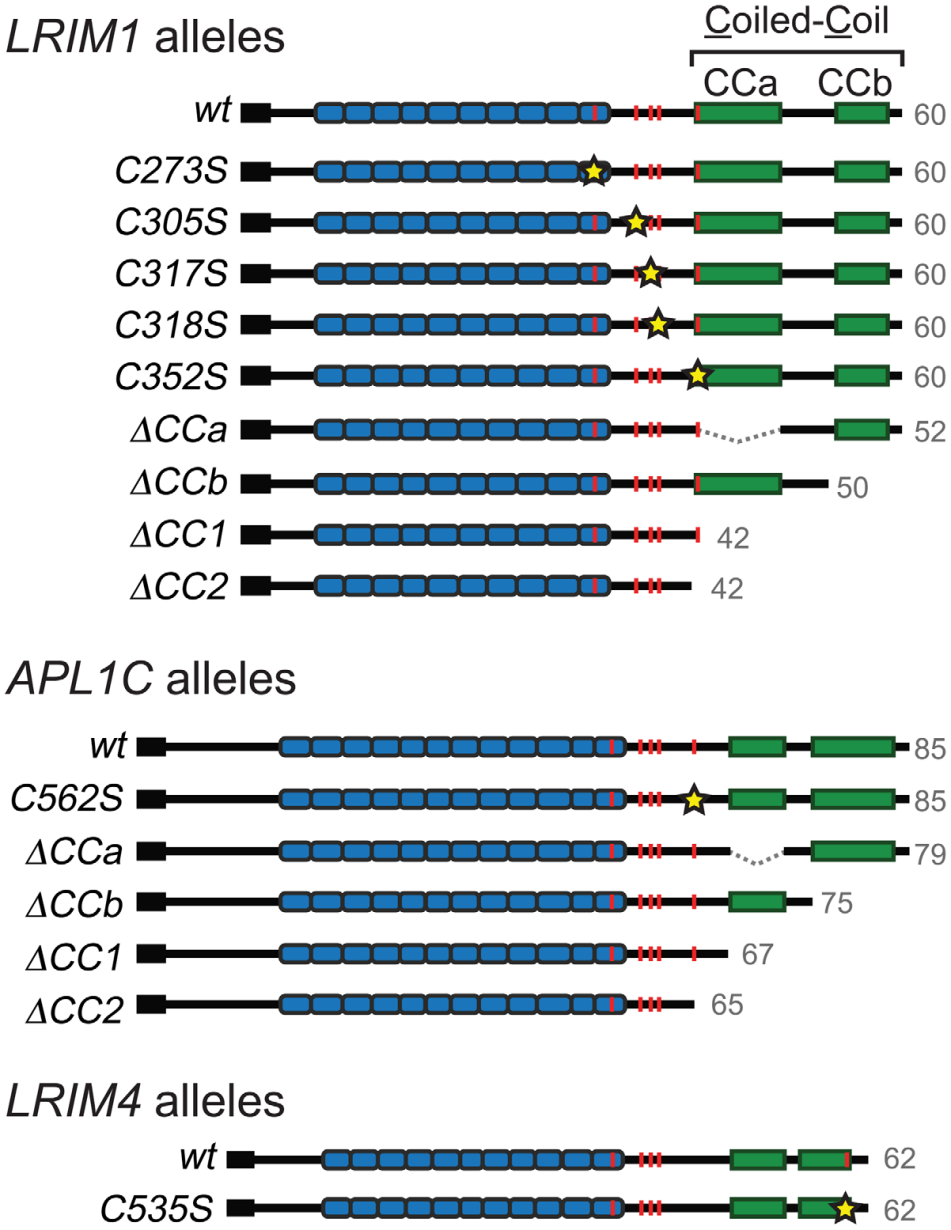
Schematic representation of LRIM1, APL1C and LRIM4 cysteine and coiled-coil alleles generated. Predicted size of the mature proteins indicated in gray. Features indicated: red line, cysteine residue; yellow star, cysteine to serine missense mutation; dashed line, in-frame deletion. Colored boxes denote: black, signal peptide; blue, LRR repeats; green, coiled-coil domains. Names of cysteine alleles indicate amino acid position of missense mutation relative to wild-type proteins. All amino acid positions are based on translation of the VectorBase mRNA sequences.

Given that the LRIM1/APL1C complex is held together by disulfide bonds, cysteine residues are implicated as a key feature in complex formation [6]. In addition, the coiled-coil domains of LRIM1 and APL1C are in direct apposition to each other in the crystal structure [15] suggesting these domains may instruct the correct assembly of LRIM1/APL1C complexes. To assay the contribution of the conserved cysteine residues and coiled-coil domain in complex formation, we engineered alleles of these features based on the *LRIM1* and *APL1C* expression constructs described above (Figure 2). Cysteine to serine missense mutations were generated for each of the 5 conserved cysteine residues of LRIM1 located between the LRR and coiled-coil domains (C273S, C305S, C317S, C318S, C352S). A single cysteine missense mutation of APL1C was generated (C562S) that targets the cysteine residue homologous to LRIM1 C352. The residue number of this cysteine in APL1C matches the *A. gambiae* PEST genome reference strain but differs from what was reported by Baxter and coworkers [15] which is likely due to a difference in the polymorphic region of PANGGL repeats [8], [12]. In addition, our previous unpublished observations indicated that LRIM4, another member of the Long subfamily of LRIM proteins, forms a disulfide-bonded homodimer. LRIM4 lacks a cysteine residue homologous to LRIM1 C352 or APL1C C562 [12]. Instead LRIM4 contains a cysteine residue (C535) at its extreme C-terminus, following the coiled-coil domain (Figure 2). We generated a missense mutation of this residue in LRIM4 (C535S) to determine whether it is responsible for homodimer formation. As LRIM1 and APL1C have bipartite coiled-coil domains with greater than 80% confidence to have the ability to form multimers (Figure S1) [19], we generated two alleles that remove each region separately (ΔCCa and ΔCCb) and two alleles that remove the coiled-coil domain altogether, one retaining LRIM1 C352 and APL1C C562 (ΔCC1) and one deleting these residues (ΔCC2) (Figure 2). Additionally, we created alternative expression constructs for LRIM1, LRIM1^C352S^, APL1C^C562S^ and APL1C^ΔCC1^ containing a C-terminal Herpes Simplex Virus (HSV) epitope tag, as some assays required proteins lacking a His tag.

### Analysis of conserved cysteine residues in LRIM1/APL1C complex formation

**Figure 3:**
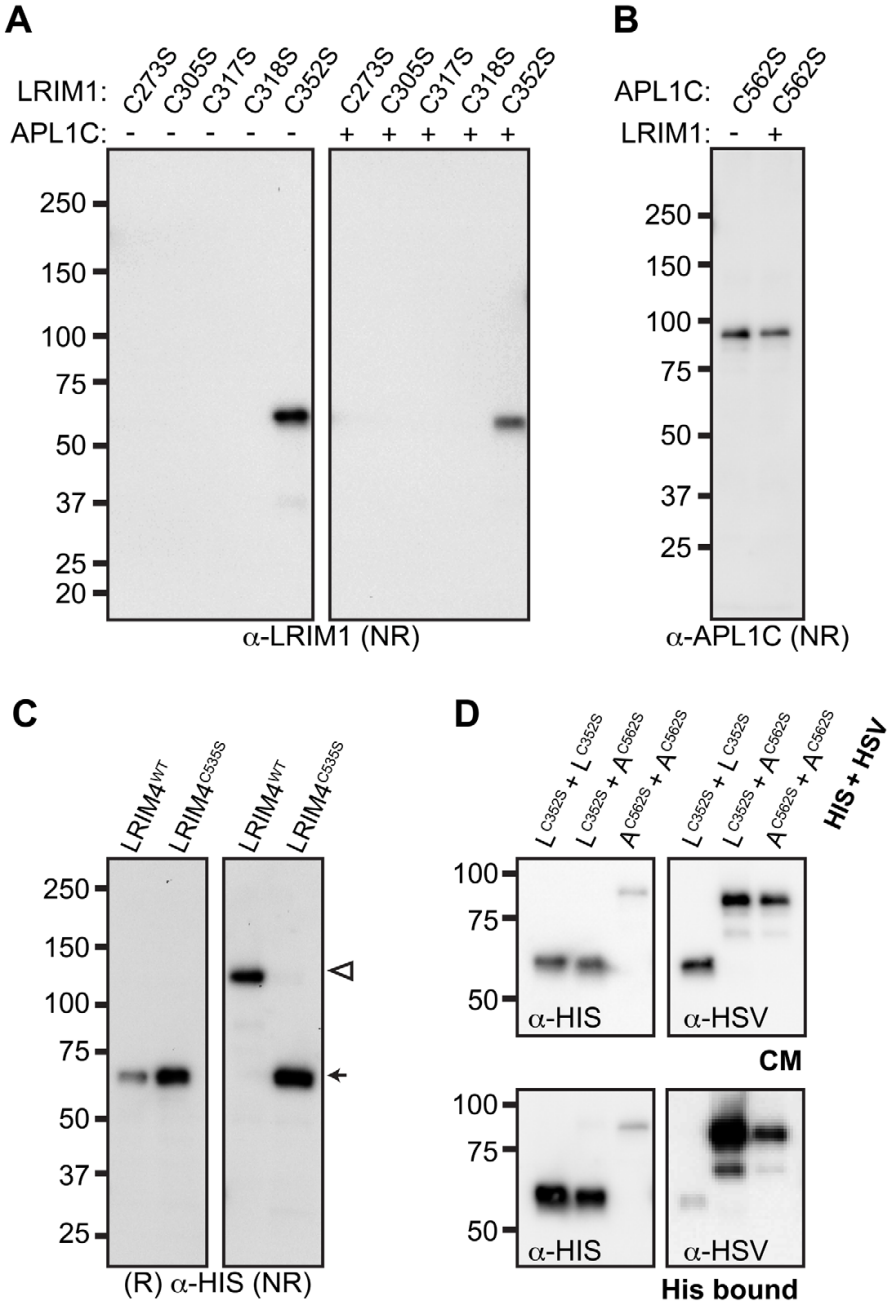
LRIM1^C352S^, APL1C^C562S^ and LRIM4^C535S^ do not form disulfide-bonded complexes. Non-reducing (NR) and reducing (R) western blot analysis of Sf9 cell CM samples following single transfection of indicated cysteine alleles (−) or co-transfection cysteine alleles together with wild-type *LRIM1* or *APL1C* (+). (**A**) Analysis of LRIM1 cysteine alleles using an antibody against LRIM1. (**B**) Analysis of APL1C^C562S^ using an APL1C antibody. (**C**) Analysis of wild-type LRIM4 and LRIM4^C535S^ using His-probe. LRIM4 monomer and homodimer are indicated by an arrow or white arrowhead, respectively. (**D**) Pull-down assays using different combinations of His- and HSV-tagged LRIM1^C352S^ (L^C352S^) and APL1C^C562S^ (A^C562S^) as indicated. The presence of the proteins was verified in the starting CM (top panels). Following His-tag capture the bound material (bottom panels) was probed for non-covalent interaction using anti-His (left panels) and anti-HSV (right panels) antibodies.

To determine which conserved cysteine residues of LRIM1 contribute to homo- and heterodimer formation, we collected samples of CM from transfected Sf9 cells and analyzed them by NR western blot. Of the 5 alleles, only *LRIM1^C352S^* produced a protein that was secreted into the CM (Figure 3A). Unlike wild-type LRIM1, which is secreted as both a monomer and homomeric complex, only monomeric LRIM1^C352S^ was present in the CM. We performed immunolocalization experiments using both LRIM1 and Strep-tag antibodies and found that all cysteine mutant proteins are produced (Figure S2). Therefore, disruption of C273, C305, C317 and C318 prevents LRIM1 secretion. We next co-expressed the *LRIM1* cysteine alleles with wild-type *APL1C* to determine if the presence of the wild-type partner would facilitate secretion or complex formation. Even when co-expressed with wild-type *APL1C*, LRIM1^C273S^, LRIM1^C305S^, LRIM1^C317S^ and LRIM1^C318S^ were still absent from the CM. Similar to when it was expressed on its own, LRIM1^C352S^ was present in the CM exclusively as a monomer. Given the importance of C352 in the ability of LRIM1 to form complexes, we tested whether a missense allele of the homologous cysteine residue (C562) of APL1C (see Figure 2) would behave similarly. We expressed *APL1C^C562S^* alone or together with wild-type *LRIM1* and found that in both cases the protein was present in the CM only as a monomer (Figure 3B). These data reveal a crucial role for the terminal conserved cysteine adjacent to the start of the coiled-coil domain of LRIM1 and APL1C in their ability to form homo- and heterodimers. To test the flexibility of the location of the cysteine involved in LRIM dimer formation, we expressed LRIM4^C535S^ in Sf9 cells. When CM was analyzed under reducing conditions, wild-type LRIM4 and LRIM4^C535S^ both migrate at approximately 62 kDa, consistent with the predicted size of a monomer (Figure 3C). When analyzed under NR conditions, LRIM4^C535S^ remains a monomer whereas wild-type LRIM4 migrates at the predicted size of a homodimer. These results indicate that LRIM4 C535 is functionally equivalent to LRIM1 C352 and APL1C C562.

### LRIM1^C352S^ and APL1C^C562S^ can form non-covalent complexes

Even though LRIM1^C352S^ and APL1C^C562S^ do not form a disulfide-linked complex, we wanted to determine whether these proteins can interact non-covalently. We co-expressed His- and HSV-tagged versions of LRIM1^C352S^ and APL1C^C562S^ in Sf9 cells. After confirming secretion of the proteins into the CM of the transfected cells (Figure 3D), we performed His pull-down experiments. Western analysis showed that the His-tagged LRIM1^C352S^ and APL1C^C562S^ were efficiently captured from the CM. Probing the captured material using an antibody against the HSV tag revealed homo- and heteromeric interactions between LRIM1^C352S^ and APL1C^C562S^ (Figure 3D). The relative strength of the observed interactions directly parallels the abilities of the wild-type proteins to homo- and heterodimerize (Figure 1); LRIM1 and APL1C interact the strongest and APL1C dimerizes more efficiently than LRIM1.

### Analysis of the coiled-coil domain in LRIM1/APL1C complex formation

**Figure 4:**
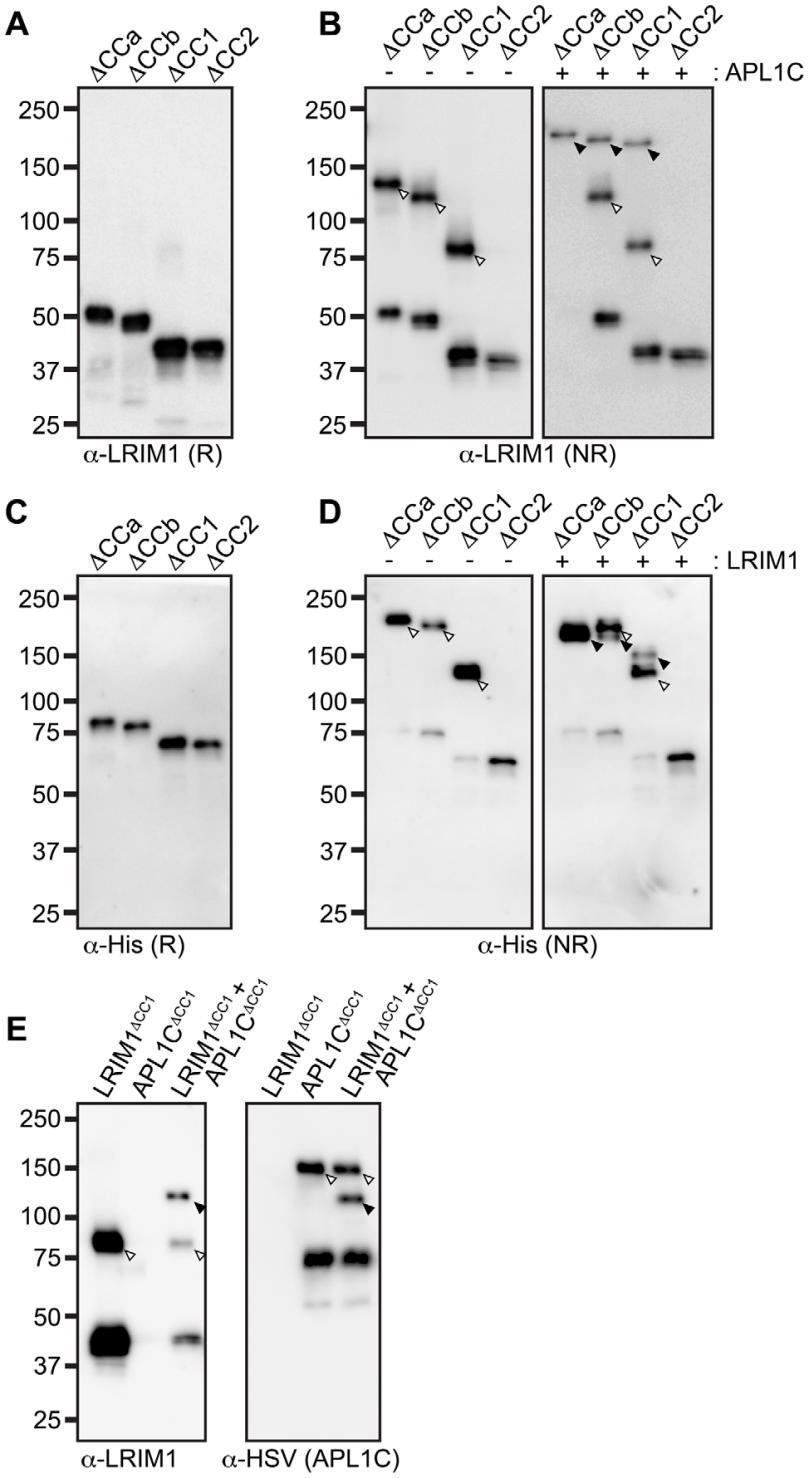
The coiled-coil domain of LRIM1 and APL1C is not required for heterodimer formation. Western blot analysis of Sf9 cell CM samples following single transfection of indicated coiled-coil alleles (−) or co-transfection of coiled-coil alleles together with wild-type *LRIM1* or *APL1C* (+). Arrowheads: black, heterodimers; white, homodimers. (**A**) Reducing (R) analysis of LRIM1 coiled-coil alleles using an antibody against LRIM1. (**B**) NR analysis of LRIM1 coiled-coil alleles transfected alone and together with wild-type APL1C. Note, there are homomeric complex bands present in the co-transfection of wild-type APL1C for both LRIM1^ΔCCb^ and LRIM1^ΔCC1^. (**C**) Reducing analysis of APL1C coiled-coil alleles using an antibody against their C-terminal His-tag. (**D**) NR analysis of APL1C coiled-coil alleles transfected alone and together with wild-type LRIM1. Note, there are homomeric complex bands present in the co-transfection of wild-type LRIM1 for both APL1C^ΔCCb^ and APL1C^ΔCC1^. (**E**) NR western analysis of LRIM1 and APL1C single and co-transfection using anti-LRIM1 (left) and anti-HSV antibodies (right) to distinguish LRIM1 and APL1C containing complexes, respectively.

To analyze how the coiled-coil domain of LRIM1 contributes to secretion and complex formation, we transfected the set of coiled-coil alleles into Sf9 cells. All of these produce proteins that are secreted into the CM in similar abundance and migrate at their expected relative sizes when analyzed under reducing conditions (Figure 4A). NR western blot analysis of the CM revealed that LRIM1^ΔCCa^, LRIM1^ΔCCb^ and LRIM1^ΔCC1^ were present as both monomers and homodimers (Figure 4B). In contrast, LRIM1^ΔCC2^, missing C352, was exclusively monomeric. Next we co-expressed the LRIM1 coiled-coil alleles with wild-type APL1C and analyzed the CM under NR conditions. The LRIM1^ΔCCa^, LRIM1^ΔCCb^ and LRIM1^ΔCC1^ proteins were each present in an additional higher molecular weight complex compared to when they were expressed alone, indicating that these proteins can form heterodimers with wild-type APL1C (Figure 4B). Again, the LRIM1^ΔCC2^ protein was only present in the CM as a monomer, demonstrating that it is unable to form complexes with itself or with APL1C. We performed a similar series of experiments with His-tagged coiled-coil alleles of *APL1C* and these behaved like their *LRIM1* counterparts. All the *APL1C* coiled-coil alleles produced proteins that were secreted into the CM and migrated at their expected sizes when analyzed under reducing conditions (Figure 4C). When analyzed under NR conditions we found that APL1C^ΔCCa^, APL1C^ΔCCb^ and APL1C^ΔCC1^ formed homodimers when expressed alone and heterodimers when co-expressed with wild-type HSV-tagged LRIM1 (Figure 4D). The APL1C^ΔCC2^ protein, missing C562, only produced a monomer both when expressed alone or when co-expressed with wild-type LRIM1 (Figure 4D).

Despite lacking a coiled-coil domain, LRIM1^ΔCC1^ and APL1C^ΔCC1^ can form homodimers when expressed alone and heterodimers when co-expressed with a wild-type partner. This suggests that the coiled-coil domain is not absolutely required for complex formation. To test this using the natural protein pair, we co-expressed His-tagged LRIM1^ΔCC1^ and HSV-tagged APL1C^ΔCC1^ to determine if a heterodimer could form between partners both lacking a coiled-coil domain. LRIM1^ΔCC1^ and APL1C^ΔCC1^ were expressed alone or together and analyzed by western blot for complex formation. As shown in Figure 4B and 4D, when expressed alone LRIM1^ΔCC1^ and APL1C^ΔCC1^ form homodimers (Figure 4E). Co-expression of LRIM1^ΔCC1^ and APL1C^ΔCC1^ produces a new complex containing both His and HSV tags that is intermediate in size to the homodimers (Figure 4E). Therefore, LRIM1^ΔCC1^ and APL1C^ΔCC1^ can form homo- and heterodimers demonstrating that the coiled-coil domain is largely dispensable for LRIM1/APL1C complex formation.

### The LRIM1/APL1C complex interacts with multiple TEP family proteins

**Figure 5:**
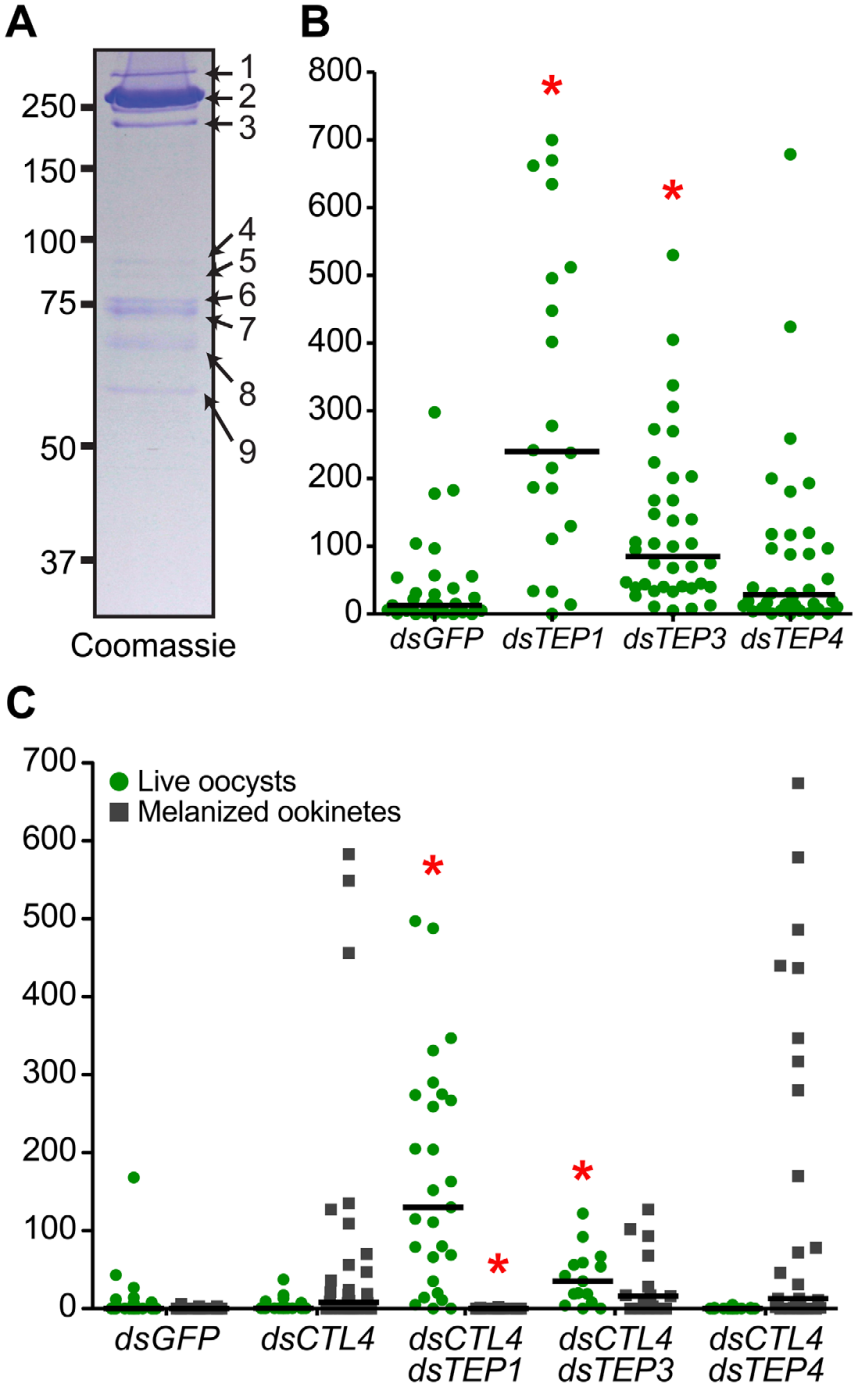
Multiple TEP family members interact with LRIM1 and APL1C. (**A**) Coomassie stained NR SDS-PAGE gel of proteins captured from CM of Sua4.0 cells transfected with His-tagged LRIM1 and APL1C. Arrows indicate protein bands analyzed by MS (also see Table 1). Note, the difference in mobility of high-molecular weight LRIM1 and APL1C complexes compared to Figure 1 is due to protein separation on 8% non-gradient gel. (**B**) Midgut oocyst numbers from mosquitoes treated with double-stranded (*ds*)*GFP*, *dsTEP1*, *dsTEP3* and *dsTEP4* RNA dissected 7 days after infection with GFP-expressing *P. berghei*. (**C**) Midgut oocyst and melanized ookinete numbers from mosquitoes treated with *dsCTL4* RNA alone or in combination with *dsTEP1*, *dsTEP3* and *dsTEP4* RNA. *dsTEP1*, *dsTEP3* and *dsTEP4* treated mosquitoes are compared to live oocyst and melanized ookinete numbers in the *dsCTL4* group. Mosquitoes treated with *dsCTL4* RNA alone have significantly fewer live oocysts and greater melanized ookinetes than *dsGFP* treated controls (P<0.0001, not indicated). Median parasite number indicated by a horizontal line and samples with significant Mann-Whitney P-values <0.0001 and <0.05 labeled with an asterisk in panels B and C, respectively.

To identify novel proteins that may function with the LRIM1/APL1C complex in parasite killing, we performed a large-scale capture experiment from the mosquito hemocyte-like cell line that was used previously to reveal an interaction between LRIM1/APL1C and the mature form of TEP1 [6]. His-tagged LRIM1 and APL1C were co-expressed and captured from the CM. Proteins co-captured with the LRIM1/APL1C complex were separated on a NR SDS-PAGE gel, visualized by staining with colloidal Coomassie and identified by mass-spectrometry (MS) (Figure 5A). The LRIM1 and APL1C homo- and heterodimers identified migrate at a slightly greater molecular weight in this assay because protein samples were separated on a fixed percentage gel (compare Figure 5A to Figure 1). In addition to LRIM1 monomer and the N- and C-terminal fragments of mature TEP1, we also identified the N- and C-terminal fragments of TEP3, the C-terminal fragment of TEP4 and the N-terminal fragment of TEP9 (Table 1). Therefore, the LRIM1/APL1C complex can interact with the mature forms of 4 different TEP proteins in the CM of mosquito cells. Band 4 at approximately 90 kDa was identified as LRIM1. Since this is too small to be a homodimer, it is probably a proteolytic fragment of a high molecular weight LRIM1 complex (band 2 or 3). A positive MS identification could not be made for band 5 that, based on molecular weight, is likely to be a monomer of APL1C. Finally, band 8 was identified as an RNA poly-A binding protein; however, given that this is an intracellular protein, it is likely to be a contaminant.

**Table 1:** Mass-spectrometry identification of proteins interacting with the LRIM1/APL1C complex.

Band	MS identification (id)	Hits
1	APL1C (AGAP007033)	4
2	LRIM1 (AGAP006438), APL1C	7, 14
3	LRIM1	3
4	LRIM1	2
5	*ND*	
6	TEP1-C (AGAP010815)	13
7	TEP1-C, TEP3-C (AGAP010816), TEP4-C (AGAP010812)	4, 8, 2
8	TEP1-N, TEP3-N, TEP9-N (AGAP010830), polyA binding protein (AGAP011092)	5, 3, 4, 3
9	LRIM1	4

Proteins bands (Figure 5A) and their gene identifier are listed together with the number of peptide hits positively identified by Mascot analysis, except for band 5, which was not determined (*ND*).

### TEP3 is an antagonist of *P. berghei*

Finding that the LRIM1/APL1C complex can interact with more than one TEP family member suggests that these TEP proteins may function in mosquito immune reactions against *Plasmodium* parasites. Interestingly, two of the TEPs identified, TEP3 and TEP4, were previously shown to play an important role in bacterial defense [3], [20]. We analyzed the role of TEP3 and TEP4 on *P. berghei* infection intensity and melanization. After *TEP3* silencing, mosquitoes showed a highly significant increase in developing oocysts 7 days post infection (Figure 5B). This increase in oocysts was not as great as upon *TEP1* silencing. In contrast, silencing *TEP4* had no effect on oocyst numbers, which is consistent with a previous report [3]. Given the important role of LRIM1, APL1C and TEP1 in parasite melanization, we next assayed whether TEP3 or TEP4 also function in this process. To test this, we silenced *TEP3* and *TEP4* together with *CTL4*, a potent inhibitor of the melanization cascade [21]. Silencing *CTL4* results in a striking increase in melanized ookinetes and a decrease in live oocysts (Figure 5C). When *TEP1* is silenced together with *CTL4,* melanization is completely blocked and there is a dramatic increase in live oocysts. Silencing of *TEP3* and *CTL4* together results in an interesting intermediate phenotype whereby there is a significant increase in oocysts but melanization is not significantly reduced (Figure 5C). *TEP4* silencing together with *CTL4* has no significant effect on oocysts numbers or parasite melanization.

### Interaction between TEP1 or TEP3 with the LRIM1/APL1C complex requires both TEP-N and TEP-C fragments

**Figure 6:**
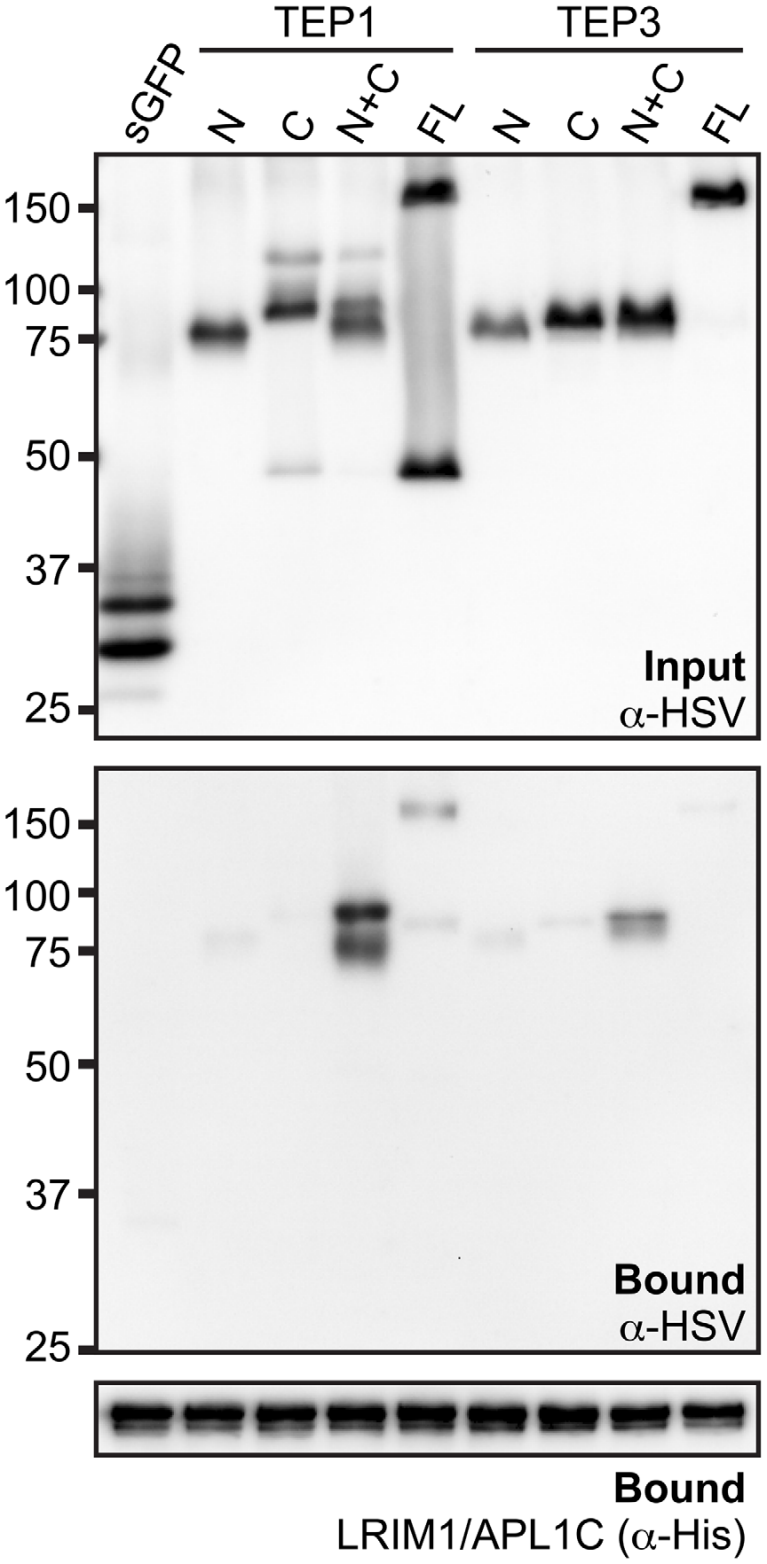
Both N- and C-terminal fragments are required for TEP1 and TEP3 interactions with the LRIM1/APL1C complex. Input CM (top panel) from Sf9 cells transfected with secreted GFP (sGFP), full-length (FL) TEP1 and TEP3 or their N- and C-terminal fragments individually (N and C) or together (N+C) was analyzed by western blot using an anti-HSV antibody. Input CM was mixed with His-tagged LRIM1/APL1C. His-bound material was probed with anti-HSV antibody (middle panel) and His-probe (lower panel).

Given that the LRIM1/APL1C complex can interact with the processed form of 4 different TEP proteins, we wanted to examine if binding is mediated by the TEP-N or TEP-C fragments independently or whether both are required. We generated HSV-tagged expression constructs for full-length TEP1 and TEP3 and their N- and C- terminal fragments. These TEPs were chosen because both TEP1 and TEP3 are *P. berghei* antagonists. CM containing HSV-tagged TEP protein fragments was mixed with CM containing His-tagged LRIM1/APL1C. Following incubation, the LRIM1/APL1C complex was captured by the His tag and samples were assayed for the presence of TEP fragments by western blot using an antibody against HSV. For both TEP1 and TEP3, we observed the strongest interaction between the LRIM1/APL1C complex and CM containing both the TEP-N and TEP-C fragments (Figure 6). Interactions between the individual fragments and full-length TEP1 and TEP3 were considerably weaker despite their similar abundance in the starting CM. These data show that the LRIM1/APL1C complex interacts strongly with TEPs only when they are processed and both N- and C- terminal fragments are present.

### TEP1 interacts with the coiled-coil domains of LRIM1 and APL1C

**Figure 7:**
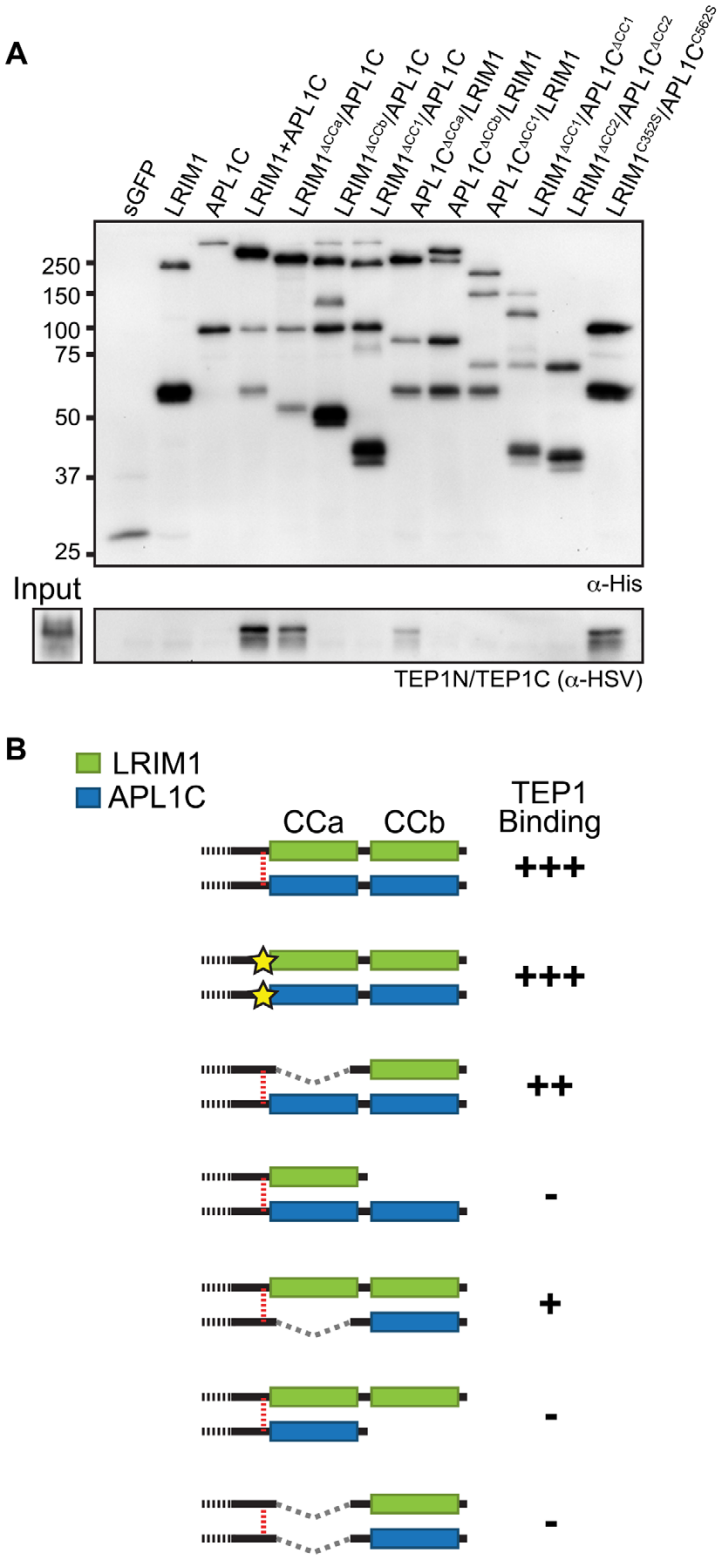
The coiled-coil CCb domain of LRIM1 and APL1C is required for TEP1 interaction. (**A**) CM containing LRIM1 and APL1C alleles indicated and the control (sGFP) were captured after mixing with input CM containing TEP1-N and TEP1-C. Capture of the LRIM1 and APL1C proteins was confirmed by western analysis of the His tag (top panel) or for TEP1-N and TEP1-C fragments using their HSV tag (bottom panel) on an 8% SDS-PAGE gel. (**B**) Summary of binding strength between LRIM1/APL1C coiled-coil complexes and TEP1-N and TEP1-C fragments. Features indicated: binding (+); no binding (-); red dashed line, disulfide bond; yellow star, cysteine to serine missense mutation; dashed line, in-frame deletion. Colored boxes denote coiled-coil ΔCCa and ΔCCb domains: green, LRIM1; blue, APL1C.

To analyze the interaction of the LRIM1/APL1C complex with TEP proteins, we performed binding assays between different LRIM1 and APL1C alleles and TEP1-N and TEP1-C. These assays aimed to reveal whether TEP1 can independently interact with both LRIM1 and APL1C and whether interaction requires disulfide-bonded complexes or an intact coiled-coil domain. We chose the Sf9 binding assay described above because this system lacks endogenous LRIMs and because in this system LRIM1 and APL1C can interact non-covalently (see Figure 3D). Following separate transfections, CM containing the LRIM1 and/or APL1C variants was mixed with CM containing TEP1-N and TEP1-C. Recombinant LRIM1 and APL1C proteins were captured using their His tag and analyzed for TEP1 binding by western blot using an HSV antibody. Strikingly, TEP1 is only captured by CM containing the LRIM1/APL1C heterodimer (Figure 7A). It was not present in samples containing only LRIM1 or APL1C monomers and homodimers.

Next we tested the ΔCCa, ΔCCb and ΔCC1 coiled-coil alleles of LRIM1 and APL1C expressed with a wild-type partner as well as co-expressed ΔCC1 and ΔCC2 alleles. Finally, given that co-expressed LRIM1^C352S^ and APL1C^C562S^ can interact non-covalently we tested whether they can cooperate to bind TEP1. We found that complexes between ΔCCa alleles with a wild-type partner captured TEP1-N and TEP1-C, but all of the other combinations of coiled-coil alleles lacked TEP1 binding despite a similar amount of captured His-tagged proteins (Figure 7A). We also observed strong binding between TEP1-N and TEP1-C when we used CM containing both LRIM1^C352S^ and APL1C^C562S^. Taken together our results, summarized in Figure 7B, demonstrate that TEP1 binds to the CCb region of the coiled-coil domain of the LRIM1/APL1C heterodimer and that this binding requires the presence of both LRIM1 and APL1C but not necessarily in a covalent complex. The coiled-coil domains of LRIM1 and APL1C are intertwined within the complex and adopt a helix-loop-helix (HLH) fold [15], which would provide ample space for protein-protein interaction. Our data reveal that the TEP1 binding site is situated within this extensive coiled-coil region of the complex and makes important contacts with the coiled-coil CCb domain.

To test the specificity of the LRIM1/APL1C interaction with TEP1, we investigated whether the LRIM4 homodimer can also interact with TEP1. We performed binding assays using the wild-type LRIM4 construct expressed in a mosquito hemocyte-like cell line described above. His-tagged LRIM4 was captured from the CM and the samples were analyzed by western blot for TEP1. No interaction was observed between LRIM4 and TEP1 (Figure S3A). As controls we expressed both His-tagged LRIM1 and APL1C, which can interact with their endogenous partner produced by these cells and capture mature TEP1. This demonstrates that the LRIM1/APL1C interaction with TEP1 is specific and not common to other LRIM dimers. Furthermore, His-tagged LRIM4 did not interact with endogenous LRIM1 or APL1C produced by these cells (data not shown). As LRIM4 does not interact with TEP1, LRIM1 or APL1C, we hypothesized that LRIM4 is not involved in *P. berghei* defense. Indeed, upon *LRIM4* knockdown there was no effect on *P. berghei* infection intensity or prevalence (Figure S3B).

## Discussion

The mosquito complement pathway plays a pivotal role in infections with *Plasmodium* parasites. In this study we biochemically dissect the structural features of the LRIM1/APL1C complex that contribute to its formation and interaction with the complement C3-like protein TEP1. LRIM1 and APL1C circulate in the adult *A. gambiae* hemolymph exclusively as a heterodimer [6]. Similarly, LRIM1 and APL1C primarily form a heterodimer when over-expressed in Sf9 or cultured mosquito cell lines. However, they can also form monomers and homodimers suggesting that these alternative forms are either highly unstable in the hemolymph or retained intracellularly due to stricter quality control mechanisms.

Numerous disulfide-bonded heterocomplexes, similar to LRIM1/APL1C, have been discovered with key roles in immunity, hemostasis and complement activation. Notable examples include the IgG heavy and light chain peptides, platelet glycoproteins Ibα and Ibβ [22] and importantly, the extensive repertoires of secreted variable lymphocyte receptor (VLR) antibodies in jawless vertebrates [23], [24]. Our study reveals that C352 of LRIM1 and the homologous residue, C562, of APL1C play a crucial role in covalently linking these proteins through a disulfide bond, which is consistent with direct apposition of these residues in the LRIM1/APL1C crystal structure [15]. Importantly, we show that this disulfide linkage is not necessary for the interaction between LRIM1 and APL1C or between the complex and the processed form of TEP1. This observation raises an important question about the role of the disulfide bond in the function of the LRIM1/APL1C complex. It is possible that the disulfide bond is necessary for the release of TEP1 from the complex during an immune response. For example, it might act as a molecular hinge and allow for a productive conformational change required for TEP1 release. Alternatively, it may increase the stability of the complex in the mosquito hemolymph and/or prevent the two proteins from acting independently, e.g. as homodimers.

The dispensability of the disulfide bridge in the formation of the LRIM1/APL1C complex also suggests that LRIM family members lacking a free cysteine residue in their C-terminal region may still be capable of forming homodimers or heterodimers. In addition, by including LRIM4 in our analysis, we demonstrate that the location of the bridging cysteine residue is flexible: despite its position on the opposite side of the coiled-coil domain, C535 of LRIM4 appears capable of forming an intermolecular disulfide bond. It remains to be seen whether the LRIM4 homodimer adopts a similar or different structure to the LRIM1/APL1C heterodimer [15]. Although *LRIM4* (also referred to as *LRRD5*) is highly upregulated in the *A. gambiae* midgut after *P. falciparum* infection [3], little is known about its functional role in the innate immune response. We demonstrate here that silencing *LRIM4* has no effect on *P. berghei* infection and that the protein does not interact with TEP1, LRIM1 or APL1C.

We show that mutations in LRIM1 of each of the remaining cysteine residues located in the region between the LRR and the coiled-coil domains (C273, C305, C317 and C318) yield proteins that are trapped within the cell and unable to be secreted into the CM even when expressed with a wild-type APL1C. Thus mutation of these cysteines is likely to grossly affect protein folding. These cysteines form two intramolecular disulfide bonds [15] and their location in all members of the LRIM protein family is at the end of the LRR domain [12]. Intramolecular disulfide bonds between adjacent cysteines in this region may generate a family-specific C-terminal cap similar to those identified in other LRR proteins [25]. Given the importance of these cysteines to the correct folding of LRIM1, it is interesting to note that the double cysteine motif in Transmembrane (TM) LRIMs is replaced by a tyrosine-cysteine pair [12]. Members of this LRIM subfamily are predicted to only form a single intramolecular bond and leaving a cysteine free to potentially form an intermolecular bridge.

Expression of various mutant or deletion LRIM1 or APL1C alleles together with their wild-type partners, respectively, implicate the cysteine-rich region as the key determinant in LRIM1/APL1C complex formation and reveal that the coiled-coil domain of these proteins is largely dispensable for heterodimer complex formation. However, we show that the most carboxy-terminal coiled-coil region (CCb) may play a role in the specificity of LRIM1 and APL1C interaction. When co-expressed with their wild-type partner, proteins lacking CCb form homo- and heterodimers with equal efficiency whereas those possessing CCb favor heterodimer formation. Importantly, the CCb region of both LRIM1 and APL1C is critical for binding to mature (processed) TEP1 altogether raising the intriguing possibility that TEPs may be involved in the specificity of complex formation.

We have revealed that the combined coiled-coil domain of the LRIM1/APL1C heterodimer is the binding site of mature TEP1 and that homodimers of LRIM1 and APL1C do not bind TEP1. In addition to revealing an interaction with mature TEP1, which was previously shown [5],[6], our MS analysis of proteins interacting with the LRIM1/APL1C complex revealed mature forms of 3 other TEP family members. It is not known whether LRIM1/APL1C interacts with each TEP individually and competitively under different circumstances. However, the reported 1∶1 stoichiometry of TEP1 to LRIM1/APL1C heterodimer [15], makes it likely that different TEPs form independent complexes with LRIM1/APL1C.

We demonstrate that one of the TEPs we found to interact with the LRIM1/APL1C complex, TEP3, is also an antagonist of *P. berghei* infections. As the increase in oocysts upon *TEP3* silencing is not as dramatic as with *LRIM1*, *APL1C* and *TEP1*, we speculate that TEP3 is either redundant or has a more indirect role in *P. berghei* killing. Another possible explanation is that TEP3 participates in a *Plasmodium* defense pathway that is distinct from or complementary to that of TEP1. This is consistent with the requirement of both TEP3 and LRIM1 for phagocytosis of gram-negative but not gram-positive bacteria whereas TEP1 is important for both [20]. Importantly, unlike TEP1, TEP3 has an inactive thioester motif [26], and although TEPs lacking an active thioester are reported to play a role in immune reactions such as phagocytosis [27] their function may be regulatory rather than structural. It remains to be determined whether TEP3 functions against the human malaria parasite, *P. falciparum*.

The LRIM1/APL1C complex also interacts with the mature form of TEP4 that plays an important role in bacterial defense and phagocytosis [3], [4], [20]. TEP4 has been previously shown to be upregulated by *P. berghei* infections [28], but we show here that it has no effect against *P. berghei*. Therefore, we hypothesize that LRIM1/APL1C and TEP4 cooperate in a defense mechanism against bacteria and that the TEP4 upregulation is due to opportunistic infections with gut bacteria that occur during *Plasmodium* traversal of the mosquito midgut epithelium. Both TEP3 and TEP4 are strongly upregulated by bacteria [29].

Our finding that the LRIM1/APL1C complex can interact with multiple members of the TEP protein family opens new avenues for investigating how mosquitoes may generate pathogen-specific immune responses. For example, LRIM1, APL1C and TEP1 have a prominent role in mosquito defense against *P. berghei*, but of these proteins only TEP1 has been shown to play a role in controlling infections with human malaria parasites, *P. falciparum* [3], [30], [31]. Just as the LRIM1/APL1C complex can interact with multiple TEP proteins, it is possible that TEP1 may also interact with multiple LRIM family members. APL1A and LRIM17 are attractive candidates given that they have been shown to be antagonists of *P. falciparum* [3], [31]. APL1A is a particularly intriguing candidate since it is 61% identical to APL1C in the coiled-coil region that contributes to the TEP1 binding site. The same region of APL1B is 78% identical to APL1C, and although APL1B has been shown to be dispensable for defense against *P. berghei* and *P. falciparum* [31], it may interact with TEP1 in a pathogen-specific manner. Future research is important to determine whether TEP1 exists in different complexes in the mosquito hemolymph and if such complexes contribute to pathogen-specific responses.

In this paper we provide the biochemical framework for understanding the role of the LRIM1/APL1C complex in regulating mosquito immunity to *Plasmodium*. Taken together, our data reveal that the LRIM1/APL1C complex is organized into three distinct modules as summarized in Figure S4. The central region containing a pattern of conserved cysteine residues is largely responsible for LRIM1/APL1C complex formation, while the coiled-coil may also contribute to the specificity of the interaction. The combined C-terminal coiled-coil region functions to carry different TEP cargoes. We show that at least four different TEP family members with distinct and overlapping roles in mosquito innate defense bind to the LRIM1/APL1C complex. Finally, we hypothesize that the LRR domains of LRIM1 and APL1C function in activation of the complex, possibly through recognition of pathogen surfaces directly or via an interaction with other immune receptors. What triggers the release of mature TEP1 from the LRIM1/APL1C complex is important to understanding how the mosquito complement pathway targets and eliminates malaria parasites.

## Materials and Methods

### Ethics statement

This study was carried out in strict accordance with the United Kingdom Animals (Scientific Procedures) Act 1986. The protocols for maintenance of mosquitoes by blood feeding and for infection of mosquitoes with *P. berghei* by blood feeding on parasite-infected mice were approved and carried out under the UK Home Office License PLL70/6347 awarded in January 2008. The procedures are of mild to moderate severity and the numbers of animals used are minimized by incorporation of the most economical protocols. Opportunities for reduction, refinement and replacement of animal experiments are constantly monitored and new protocols are implemented following approval by the Imperial College Ethical Review Committee.

### Generation of *LRIM1*, *APL1C* and *LRIM4* alleles

Alleles of *LRIM1*, *APL1C* and *LRIM4* were generated by PCR using *pIEx10* clones as templates and primers listed in the Table S1. Products were LIC cloned (Merck Chemicals) into a dual Strep- and His-tag vector (*pIEx10*) or InFusion cloned (Clontech) into an HSV-tag (*pIEx1SPmyc*) vector. The *pIEx1SPmyc* vector is a variant of the *pIEx1* (Merck Chemicals) that retains its C-terminal HSV tag but was modified to contain an N-terminal IgM signal peptide and Myc epitope tag. Cysteine and ΔCCa alleles of *LRIM1* and *APL1C* were generated by splice-overlap extension (SOE) PCR [32] using outer “His” and inner allele-specific primers. The *LRIM4^C535S^* missense mutation was created using the QuikChange II Site-Directed Mutagenesis protocol (Stratagene). All constructs were verified by DNA sequencing.

### Western blot conditions

Protein samples were separated on 8% or 4-15% Criterion SDS-PAGE gels (Bio-Rad). NR samples were prepared in Lane Marker sample buffer (Pierce). Reduced samples were made by supplementing NR samples with a tris(2-carboxyethyl)phosphine (TCEP) solution (Pierce) to a final concentration of 25 mM and heating at 95°C for 5 min. Transfer to PVDF and western conditions were previously described [6] except for rabbit α-GFP (1∶1000 diluted in PBS + 0.05% Tween 20 and 3% milk), mouse α-Strep-tag (1∶200) and goat α-HSV-tag (1∶1000) diluted in PBS + 0.05% Tween 20 and 3% BSA.

### Protein expression, purification and mass spectrometry

Analysis of *LRIM1*, *APL1C* and *LRIM4* alleles was performed using the CM of transfected Sf9 cells adapted to growth in serum-free (SFM) culture medium (Sf-900 II, Invitrogen). Cells were transfected using Escort IV reagent (Sigma) and CM was collected 3-4 days post transfection, cleared of debris by centrifugation or by passage through a 0.45 µm filter and supplemented with NR sample buffer. Unless noted, a 2∶1 µg ratio of APL1C to LRIM1 was used in all co-transfection experiments to achieve comparable expression levels. Hemolymph was collected as described previously [6]. For MS analysis, mosquito Sua4.0 cells were transfected using Effectene (Qiagen) at 80–90% confluence in non-vented 175 mm^2^ culture flasks. DNA complexes were made by diluting 7.5 µg of *pIEx10-LRIM1* and 17.6 µg *pIEx10-APL1C* with 1.3 mL EC buffer and then adding 40 µL of enhancer followed by 125 µL of Effectene reagent. Complexes were added to cells dropwise and incubated for 12 h in Schneider's *Drosophila* (S2) medium containing 10% heat-inactivated FCS. Cells were washed with and then placed in 30 mL of serum-free S2 medium for conditioning. CM was collected after 5 days, passed through a 0.45 µm syringe filter into a conical tube and supplemented with 0.05% triton X-100. A slurry of 650 µL (packed volume) of Ni-NTA agarose (Qiagen) in PBS was added to the CM and mixed for 2 h at room temperature. Beads were washed once in the tube with buffer (50 mM NaH_2_PO_4_, 300 mM NaCl and 20 mM imidazole pH 8.0) and then transferred to a 10 mL disposable column for further washes. Bound proteins were eluted in 10 mL of wash buffer containing 250 mM imidazole and then concentrated to approximately 100 µL using a 10 kDa cutoff Amicon Ultra filter (Millipore). Samples were made by the addition of NR buffer, separated on an 8% SDS-PAGE gel, stained with Imperial stain (Pierce) and imaged before MS identification of individual bands (performed at EMBL).

### Binding assays

TEP1 binding assays of *LRIM1* and *APL1C* alleles were performed using the CM of transfected Sf9 cells. Cells were transfected independently with *pIEx1SPmyc-TEP* and *pIEx10-APL1C*/*pIEx10*-*LRIM1* vectors. 200 µL of each CM was mixed and supplemented with 0.1% triton X-100 and 5 µL of a 1∶1 slurry of Talon resin (Stratagene) in PBS. After a 3 h incubation at room temperature the beads were washed in PBS containing 0.1% triton X-100 and extracted with 35 µL of 2x NR loading buffer. Binding between LRIM1^C352S^ and APL1C^C562S^ was determined by performing His pull-down assays using CM from Sf9 cells co-transfected with *pIEx10-LRIM1^C352S^* and *pIEx1SPmyc-APL1C^C562S^* plasmids, respectively. TEP1 binding assays for wild-type LRIM4 were performed using the CM of transfected mosquito Sua4.0 cells as described previously [6].

### Mosquito maintenance, gene silencing and infection

The N'gousso strain of *A. gambiae* was maintained as described previously [33], [34]. Mosquitoes were cultured and infected with *P. berghei* CON_GFP_ strain [35] as described previously [11]. Single and double knockdown experiments and parasite counts in dissected midguts were performed as described previously [6]. Primers used for synthesis of double stranded RNA are listed in Table S1.

### VectorBase gene identifiers

LRIM1, AGAP006348; APL1C, AGAP007033; LRIM4, AGAP007039; TEP1, AGAP010815; TEP3, AGAP010816; TEP4, AGAP010812; TEP9, AGAP010830; polyA-binding protein, AGAP011092; S7, AGAP010592; CTL4, AGAP005335.

## Supporting Information

Figure S1 **Probability of coiled-coil and coiled-coil multimer formation in LRIM1 and APL1C.** (**A**) Potential for coiled-coil formation as a function of amino acid position [19]. Red double arrow indicates the region between the coiled-coil CCa and CCb domains (black double arrow) with very low probability of coiled-coil formation. (**B**) Potential for coiled-coil multimers [36]. Lines indicated: green, dimer probability; blue, trimer probability; gray with shaded area underneath, total multimer probability (dimers and trimers); Dashed line indicates the 80% threshold.
(TIF)

Figure S2 **LRIM1 cysteine allele protein expression in transfected Sf9 cells.** One day after transfection with indicated *LRIM1* cysteine alleles, cells were fixed, permeabilized and labeled with GFP or LRIM1 (red, middle panels) or Strep-tag (green, top panels) antibodies. DIC images (bottom panels) show an approximately equal number of cells were analyzed for each allele. The scale for all pictures is identical. Scale bar in the top left panel is 20µm.
(TIF)

Figure S3 **LRIM4 does not interact with TEP1 and does not affect** ***P. berghei*** **development.** (**A**) CM input (left panels) and His-captured samples (right panels) from Sua4.0 cells transfected with sGFP, LRIM4, APL1C and LRIM1 analyzed by NR western blot using the His-tag (top panels) or an antibody against TEP1 (bottom panels). (**B**) Midgut oocyst numbers from mosquitoes treated with *dsGFP*, *dsLRIM4* and *dsLRIM1* RNA dissected 7 days after infection with GFP-expressing *P. berghei*. Prevalence of infection was 91%, 96% and 100% for *dsGFP*, *dsLRIM4* and *dsLRIM1*, respectively. Median parasite number indicated by a horizontal line and samples with significant Mann-Whitney P-values (<0.0001) labeled with an asterisk.
(TIF)

Figure S4 **The modular organization of the LRIM1/APL1C heterodimer.** Schematic representation of the LRIM1/APL1C complex with its 3 modules highlighted.
(TIF)

Table S1 **Primer sequences.** Primers used for generation of dsRNA, qRT-PCR and protein expression constructs. For LIC cloning into *pIEx10* His F primers have GACGACGACAAGATG and His R primers have GAGGAGAAGCCCGGTTT at their 5′ end indicated by a * symbol. For InFusion cloning into *pIEx1SPmyc* HSV F primers have TACCGGTTCGAAGCTT and HSV R primers have GTGCGGCCGCAAGCTT at their 5′ end indicated by a # symbol. SOE PCR fragments generated by primer pairs His F/f1 R and f2 F/His R were mixed and used as a template in a reaction with His F/His R to generate full-length products.
(DOC)
