# Supplementary figures and images for: Structure-Function Analysis of the Anopheles gambiae LRIM1/APL1C Complex and its Interaction with Complement C3-Like Protein TEP1
Source: PLoS Pathog. 2011 Apr 14;7(4):e1002023. doi: 10.1371/journal.ppat.1002023 (PMC3077365; doi:10.1371/journal.ppat.1002023)

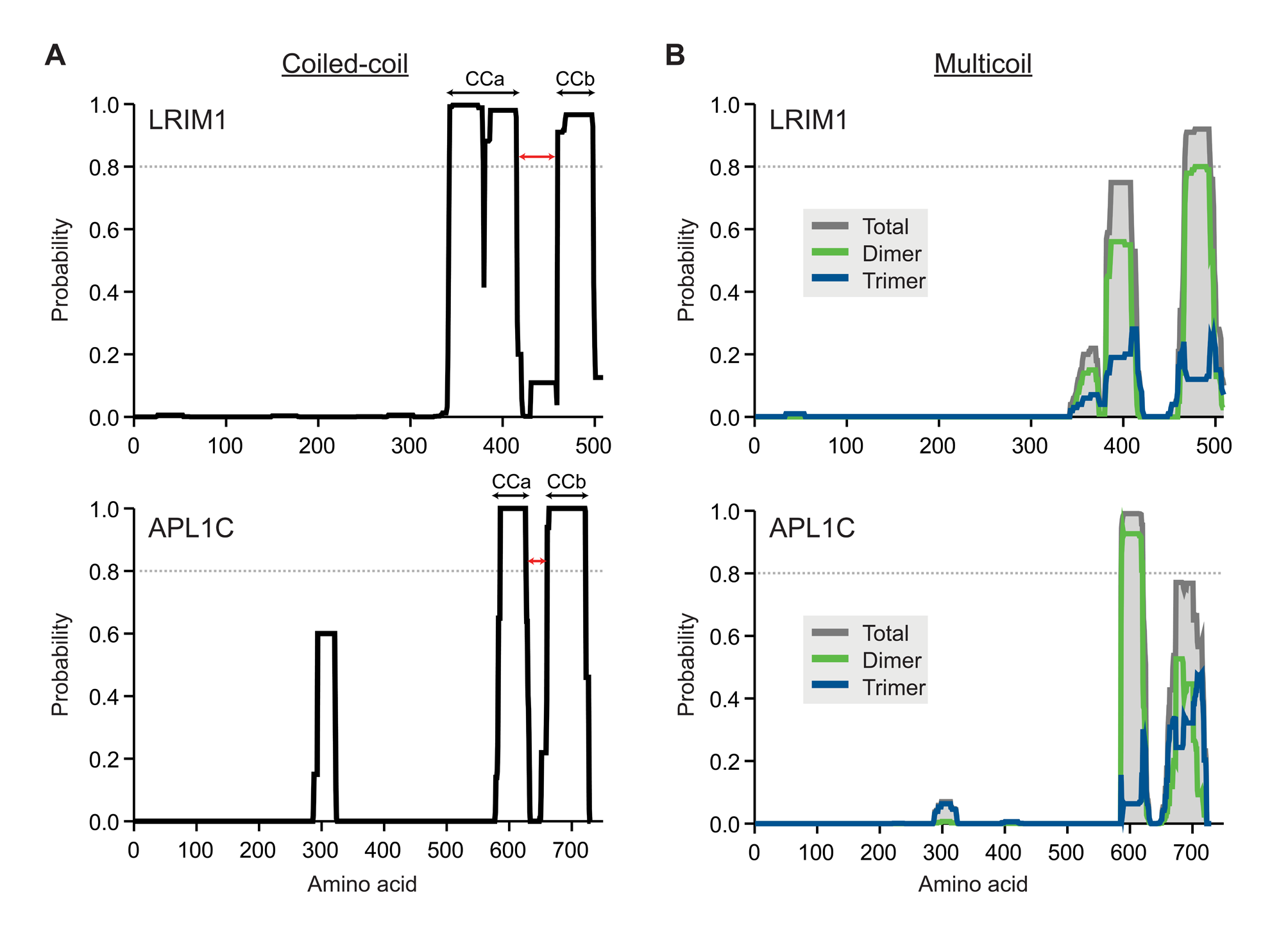

Supplement: Figure S1 — Probability of coiled-coil and coiled-coil multimer formation in LRIM1 and APL1C. (A) Potential for coiled-coil formation as a function of amino acid position [19]. Red double arrow indicates the region between the coiled-coil CCa and CCb domains (black double arrow) with very low probability of coiled-coil formation. (B) Potential for coiled-coil multimers [36]. Lines indicated: green, dimer probability; blue, trimer probability; gray with shaded area underneath, total multimer probability (dimers and trimers); Dashed line indicates the 80% threshold. (TIF) [file ppat.1002023.s001.tif]

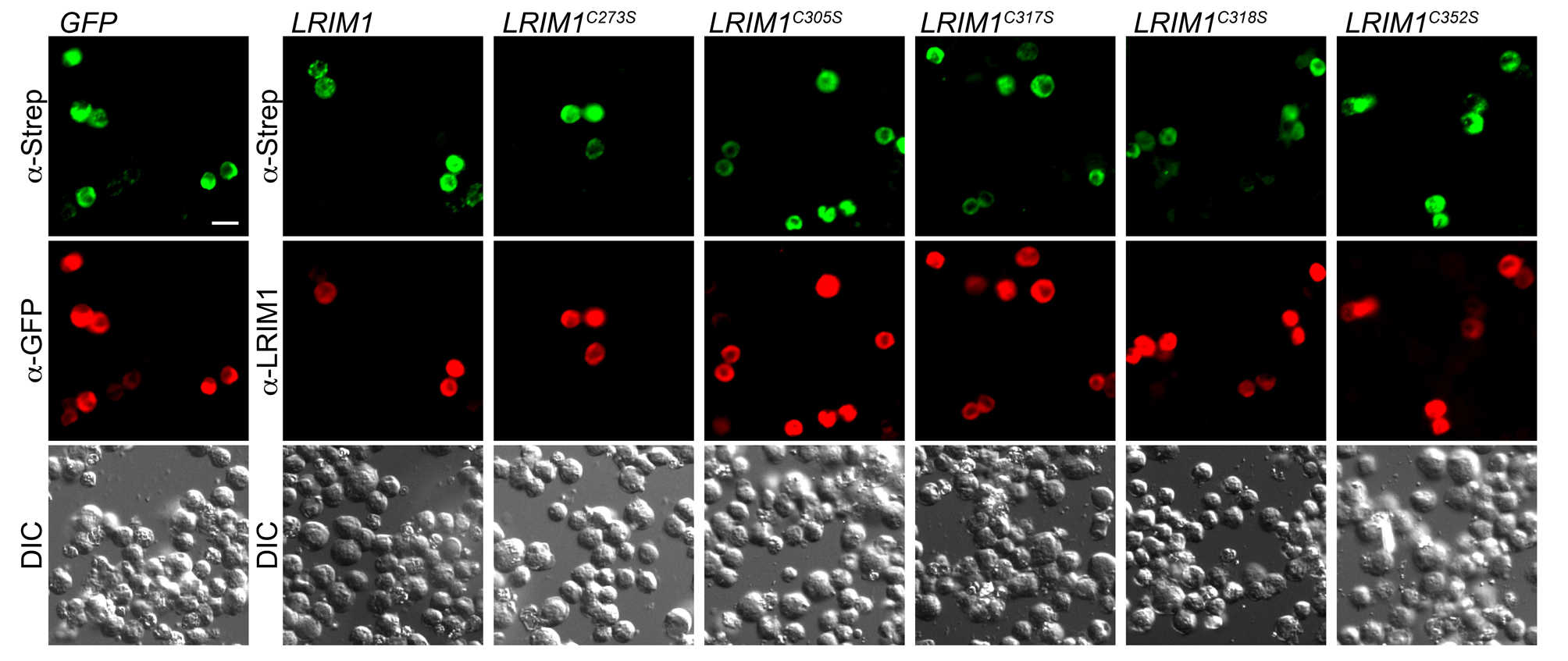

Supplement: Figure S2 — LRIM1 cysteine allele protein expression in transfected Sf9 cells. One day after transfection with indicated LRIM1 cysteine alleles, cells were fixed, permeabilized and labeled with GFP or LRIM1 (red, middle panels) or Strep-tag (green, top panels) antibodies. DIC images (bottom panels) show an approximately equal number of cells were analyzed for each allele. The scale for all pictures is identical. Scale bar in the top left panel is 20µm. (TIF) [file ppat.1002023.s002.tif]

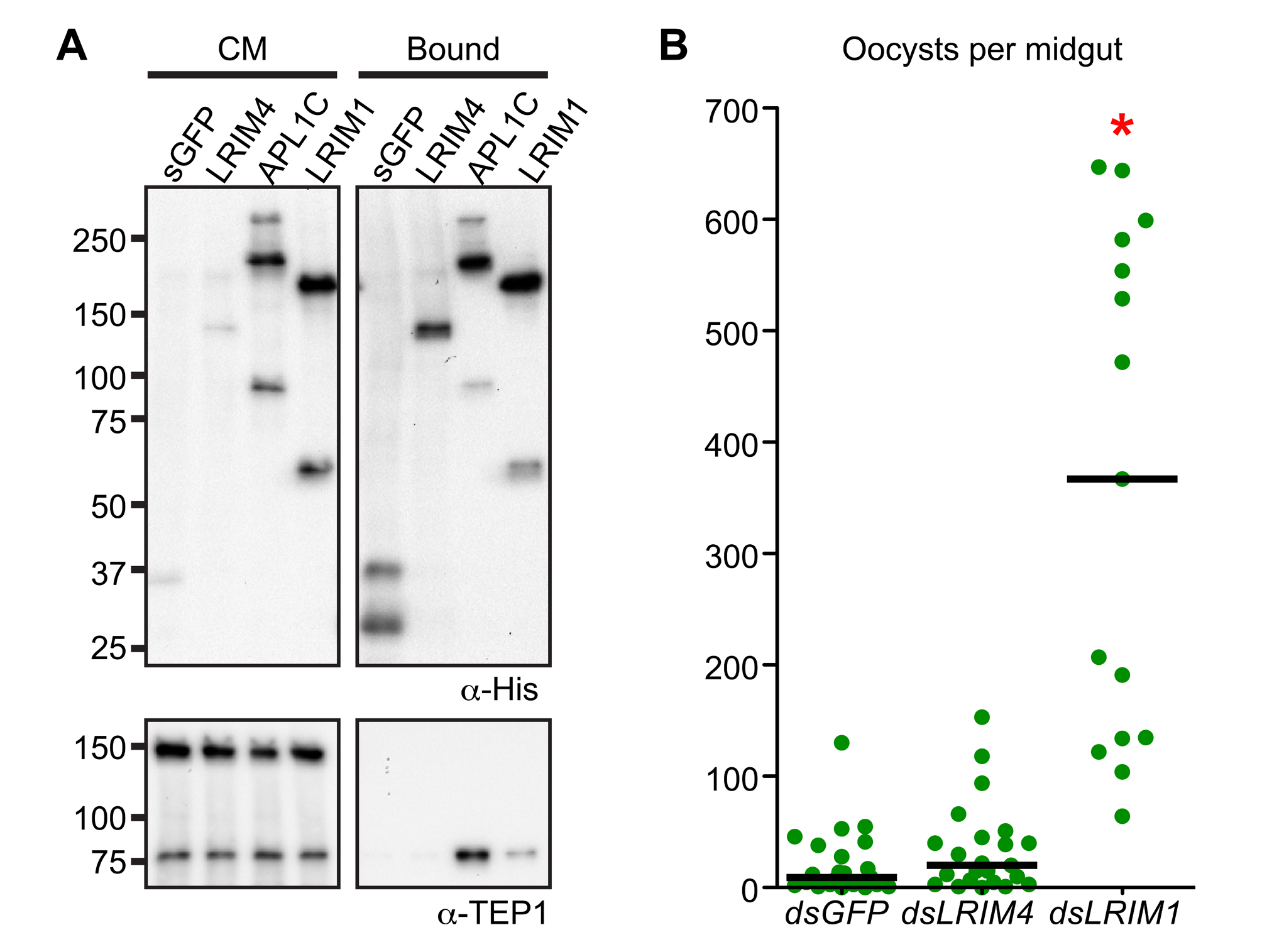

Supplement: Figure S3 — LRIM4 does not interact with TEP1 and does not affect P. berghei development. (A) CM input (left panels) and His-captured samples (right panels) from Sua4.0 cells transfected with sGFP, LRIM4, APL1C and LRIM1 analyzed by NR western blot using the His-tag (top panels) or an antibody against TEP1 (bottom panels). (B) Midgut oocyst numbers from mosquitoes treated with dsGFP, dsLRIM4 and dsLRIM1 RNA dissected 7 days after infection with GFP-expressing P. berghei. Prevalence of infection was 91%, 96% and 100% for dsGFP, dsLRIM4 and dsLRIM1, respectively. Median parasite number indicated by a horizontal line and samples with significant Mann-Whitney P-values (<0.0001) labeled with an asterisk. (TIF) [file ppat.1002023.s003.tif]

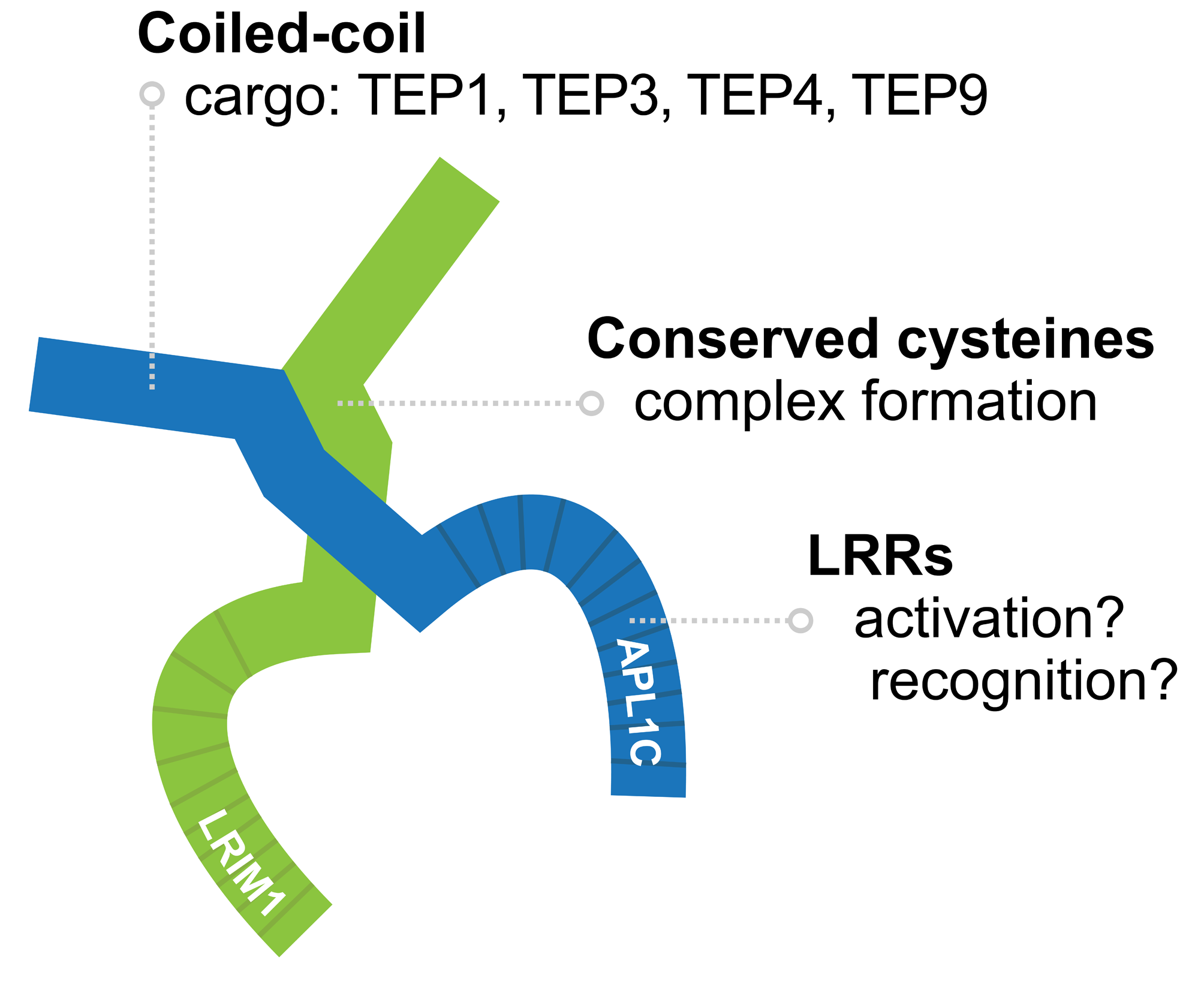

Supplement: Figure S4 — The modular organization of the LRIM1/APL1C heterodimer. Schematic representation of the LRIM1/APL1C complex with its 3 modules highlighted. (TIF) [file ppat.1002023.s004.tif]
