# Supplementary material for: Structure-Function Analysis of the Anopheles gambiae LRIM1/APL1C Complex and its Interaction with Complement C3-Like Protein TEP1
Source: PLoS Pathog. 2011 Apr 14;7(4):e1002023. doi: 10.1371/journal.ppat.1002023 (PMC3077365; doi:10.1371/journal.ppat.1002023)
Supplement: Table S1 — Primer sequences. Primers used for generation of dsRNA, qRT-PCR and protein expression constructs. For LIC cloning into pIEx10 His F primers have GACGACGACAAGATG and His R primers have GAGGAGAAGCCCGGTTT at their 5′ end indicated by a * symbol. For InFusion cloning into pIEx1SPmyc HSV F primers have TACCGGTTCGAAGCTT and HSV R primers have GTGCGGCCGCAAGCTT at their 5′ end indicated by a # symbol. SOE PCR fragments generated by primer pairs His F/f1 R and f2 F/His R were mixed and used as a template in a reaction with His F/His R to generate full-length products. (DOC) [file ppat.1002023.s005.doc]

| **Primers for LRIM1 alleles** | |
| --- | --- |
| LRIM1^His^ F | *GCAATACACGAGATAAAGCAG |
| LRIM1^His^ R | *TCCCAGCTGGCTCGCTAAATTC |
| LRIM1^HSV^ F | #GCAATACACGAGATAAAGCAG |
| LRIM1^HSV^ R | #TCCCAGCTGGCTCGCTAAATTC |
| LRIM1^C273S^ f1 R | CACGCAACGTTCCAGAGTGAAACCCGTTG |
| LRIM1^C273S^ f2 F | CAACGGGTTTCACTCTGGAACGTTGCGTG |
| LRIM1^C305S^ f1 R | CGTTGGCACGGTAGACTCTTCCTCATTC |
| LRIM1^C305S^ f2 F | GAATGAGGAAGAGTCTACCGTGCCAACG |
| LRIM1^C317S^ f1 R | CAGATCCTCGCAGGAGTACGCCCCATAG |
| LRIM1^C317S^ f2 F | CTATGGGGCGTACTCCTGCGAGGATCTG |
| LRIM1^C318S^ f1 R | CGGCAGATCCTCGGAGCAGTACGCCCC |
| LRIM1^C318S^ f2 F | GGGGCGTACTGCTCCGAGGATCTGCCG |
| LRIM1^C352S^ f1 R | GTTTTCCCGCTCAGACTCGAGCCGCTC |
| LRIM1^C352S^ f2 F | GAGCGGCTCGAGTCTGAGCGGGAAAAC |
| LRIM1^ΔCCa^ f1 R | CTGCAGCTCGATACACTCGAGCCGCTCCGTTTC |
| LRIM1^ΔCCa^ f2 F | CGGCTCGAGTGTATCGAGCTGCAGCACGCGACC |
| LRIM1^ΔCCb-His^ R | *CTCGGTCGCGTGCTGCAGCTC |
| LRIM1^ΔCC1-His^ R | *CTGGTTTTCCCGCTCACACTC |
| LRIM1^ΔCC2-His^ R | *CTCGAGCCGCTCCGTTTCCGAG |
| **Primers for APL1C alleles** | |
| APL1C^His^ F | *GTTGGGGGCAATAATTATTGG |
| APL1C^His^ R | *TGTAACGCGACGCGTATCTGG |
| APL1C^HSV^ F | #GTTGGGGGCAATAATTATTGG |
| APL1C^HSV^ R | #TGTAACGCGACGCGTATCTGG |
| APL1C^C562S^ f1 R | GTATCTGTGGCACTGGAGCGTCCTTGTGC |
| APL1C^C562S^ f2 F | GCACAAGGACGCTCCAGTGCCACAGATAC |
| APL1C^ΔCCa^ f1 R | CAGGCGGTACCGCTGTGTGATGTAGTGGGACAG |
| APL1C^ΔCCa^ f2 F | CTACATCACACAGCGGTACCGCCTGCCGAAGGATGG |
| APL1C^ΔCCb-His^ R | *ATTTAGATTATCGCTCGAGCG |
| APL1C^ΔCC1-His^ R | *TGTGATGTAGTGGGACAGACTTTG |
| APL1C^ΔCC1-HSV^ R | #TGTGATGTAGTGGGACAGACTTTG |
| APL1C^ΔCC2-His^ R | *GCGTCCTTGTGCACGCTGCAAC |
| **Primers for LRIM4 alleles** | |
| LRIM4^His^ F | *AAGCCATTGCAGTTTGCGTGC |
| LRIM4^His^ R | *CTGAATAATGACCGTTTGTCC |
| LRIM4^C535S^ QC F | GCAGCTGACCAAGAGCACCTCGACCGT |
| LRIM4^C535S^ QC R | ACGGTCGAGGTGCTCTTGGTCAGCTGC |
| **Primers for TEP1 and TEP3 expression** (Full-length clones use TEPN F and TEPC R) | |
| TEP1N^HSV^ F | #CTACTGGTTGTGGGTCCGAAATTTATACG |
| TEP1N^HSV^ R | #GCCTGACTGCAGTGCATTACGCCC |
| TEP1C^HSV^ F | #ACGGGGCGTAATGCACTGCAGTCAGG |
| TEP1C^HSV^ R | #CTTGCACTCTGCGGGACAGTCTTC |
| TEP3N^HSV^ F | #GTGCTGGTGGTAGGTCCGAAATTTG |
| TEP3N^HSV^ R | #GGAAGCTGCGCTACCGAAGCGCTC |
| TEP3C^HSV^ F | #GAGCGCTTCGGTAGCGCAGCTTCCCG |
| TEP3C^HSV^ R | #GTCGAGGTAGCTCTGGATATCTGC |
| **Primer for dsRNA** (all have TAATACGACTCACTATAGGG sequence on their 5' end) | |
| dsGFP F | ACGTAAACGGCCACAAGTTC |
| dsGFP R | TGTTCTGCTGGTAGTGGTCG |
| dsCTL4 F | TGGTTTGATGCCGTGTCCT |
| dsCTL4 R | AATAAATTGTCTCGGTTCATCATC |
| dsTEP1 F | TTTGTGGGCCTTAAAGCGCTG |
| dsTEP1 R | ACCACGTAACCGCTCGGTAAG |
| dsTEP3 F | CGAGAAGGAACCCATTTAAGG |
| dsTEP3 R | GCTGCTGGAATGGCATAAGT |
| dsTEP4 F | TCTTCTGGGAGGATGTTTGG |
| dsTEP4 R | ACGGTGGTCAATTGAAGAGG |
| dsLRIM4 F | ATCTGGAGCTGCACGAAAAT |
| dsLRIM4 R | CCCCTCCTGAAGGCTTTTAC |
| **Primers for qRT-PCR** | |
| qRT TEP1 F | AAAGCTGTTGCGTCAGGG |
| qRT TEP1 R | TTCTCCCACACACCAAACGAA |
| qRT TEP3 F | GGAAAGCATTGCGGATGTAT |
| qRT TEP3 R | TTGGTAGCGATTCCCAGTTC |
| qRT TEP4 F | GCTGAAGGCACTTACCAAGC |
| qRT TEP4 R | CGCGAAACTCTTTCTTACGG |
| qRT S7 F | GTGCGCGAGTTGGAGAAGA |
| qRT S7 R | ATCGGTTTGGGCAGAATGC |
| qRT LRIM4 F | CTGTTTACCGTGCAGACCAC |
| qRT LRIM4 R | AGCACGGTCAGGAAGTTGTT |
